# Transformation of 2-Line Ferrihydrite to Goethite at Alkaline pH

**DOI:** 10.1021/acs.est.3c05260

**Published:** 2023-10-12

**Authors:** Fabio E. Furcas, Barbara Lothenbach, Shishir Mundra, Camelia N. Borca, Cristhiana Carine Albert, O. Burkan Isgor, Thomas Huthwelker, Ueli M. Angst

**Affiliations:** † Institute for Building Materials, ETH Zürich, 8093 Zürich, Switzerland; ‡ Empa Concrete & Asphalt Labortory, 8600 Dübendorf, Switzerland; § Swiss Light Source, Paul Scherrer Institut, 5232 Villigen, Switzerland; ∥ School of Civil and Construction Engineering, Oregon State University, Corvallis, 97331 Oregon, United States

**Keywords:** precipitation, iron, kinetics, pH, XAS, XRD, ICP, TGA

## Abstract

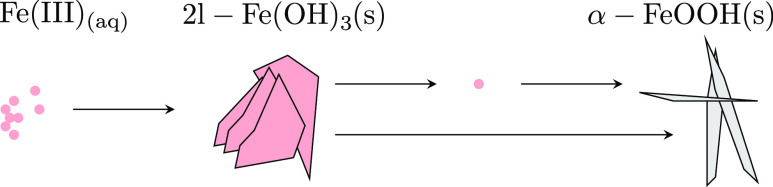

The transformation of 2-line ferrihydrite to goethite from supersaturated solutions at alkaline pH ≥ 13.0 was studied using a combination of benchtop and advanced synchrotron techniques such as X-ray diffraction, thermogravimetric analysis, and X-ray absorption spectroscopy. In comparison to the transformation rates at acidic to mildly alkaline environments, the half-life, *t*_1/2_, of 2-line ferrihydrite reduces from several months at pH = 2.0, and approximately 15 days at pH = 10.0, to just under 5 h at pH = 14.0. The calculated-first order rate constants of transformation, *k*, increase exponentially with respect to the pH and follow the progression log_10_*k* = log_10_*k*_0_ + *a*·pH^3^. Simultaneous monitoring of the aqueous Fe(III) concentration via inductively coupled plasma optical emission spectroscopy demonstrates that (i) goethite likely precipitates from solution and (ii) its formation is rate-limited by the comparatively slow redissolution of 2-line ferrihydrite. The analysis presented can be used to estimate the transformation rate of naturally occurring 2-line ferrihydrite in aqueous electrolytes characteristic to mine and radioactive waste tailings as well as the formation of corrosion products in cementitious pore solutions.

## Abstract 2

Limited research exists on the transformation of 2-line ferrihydrite at alkaline pH. This study reveals a significant reduction in its half-life, impacting its effectiveness in immobilizing toxic elements.

## Introduction

Amorphous Fe(III) (hydr)oxide intermediates and end members can be ranked according to their thermodynamic stability. 2-Line ferrihydrite (2l-Fe(OH)_3_(s), bulk composition Fe_10_O_14_(OH)_2_), a nanocrystalline iron hydroxide, is generally recognized as the least stable naturally occurring iron-bearing phase. The absence of a long-range order of arrangement renders it thermodynamically unstable compared to other, more crystalline iron (hydr)oxides. Due to its remarkable sorption capacity and high surface area,^1−3^ 2-line ferrihydrite readily adsorbs groundwater contaminants^4−6^ and is commonly used in a range of industrial applications including heavy metal sequestration and nitrogen removal from wastewater.^7−9^ Its formation and transformation to other iron (hydr)oxides dictate the amount of iron that remains mobile in the aqueous phase, prospectively impacting the long-term performance of nuclear waste repositories and reinforced concrete structures.^10−15^ Moreover, iron is present in significant quantities (>1 g L^–1^) in uranium mill raffinates.^16^ Precipitation as amorphous Fe(OH)_3_(s), ferric arsenate, and hydrotalcite/layered double hydroxide and subsequent adsorption of As are an important mechanism controlling the solubility of elements of concern (EOCs) during raffinate neutralization.^17−19^ Since most of the highly crystalline ferric (hydr)oxide phases can coexist with or be synthesized from 2-line ferrihydrite,^2,20−22^ it can be considered a gateway compound that plays an important role in the kinetic mechanism leading to their formation.

Phase transformation of 2-line ferrihydrite to hematite and goethite has been investigated extensively at low (pH = 2) to moderately alkaline (pH = 12) pH and temperatures ranging from 4 to 100 °C.^1,20−32^ While goethite is preferentially stabilized at low to moderate temperatures and either acidic (pH ≤ 6) or alkaline (pH ≥ 10) conditions,^20,23,29^ the formation of hematite is favored at circumneutral pH or elevated temperatures.^25,28,29,31^ In the former case, rapid dissolution of 2-line ferrihydrite prompts reprecipitation into goethite from aqueous Fe(OH)^2+^ at slightly acidic and Fe(OH)_4_^–^ at alkaline pH.^1,24^ As dissolution of 2-line ferrihydrite is minimal at circumneutral pH, its transformation into hematite proceeds via a two-step crystallization process with goethite forming as an intermediate.^1,22,28,29,31,32^ Depending on the presence of Cl^–^, HCO_3_^–^, and SO_4_^2–^, the formation of lepidocrocite as an intermediate is observed prior to or concomitant with the formation of goethite during the Fe(II)-induced transformation of ferrihydrite under anoxic conditions.^33^

Irrespective of the stabilized end member, transformation rates appear to follow first-order kinetics with respect to the amount of 2-line ferrihydrite present.^23,24,29^ The formation of either hematite or goethite is said to be dissolution-controlled. Studies furthermore suggest that the rate of transformation is strictly related to the amount of OH^–^ present in the system. At acidic pH, this observation is in keeping with the thermodynamic and kinetic aspects constituting the crystallization process: first, the solubility limit, as dictated by the dissolution of any iron (hydr)oxide species, shows a minimum at the point of zero charge (PZC)^23^ at ∼pH 8. An increase in the pH from acidic toward circumneutral conditions thus increases the thermodynamic driving force for primary nucleation of goethite or hematite to occur. Second, Fe(III) precipitation proceeds more rapidly if the aqueous OH^–^ to Fe ratio mimics that of the solid phase stabilized.^20,34,35^ At alkaline pH, however, the solubility limit of Fe(III) coincides with the concentration of Fe(OH)_4_^–^, which increases by about 1 order of magnitude per unit increase in pH.^36^ High levels of alkalinity beyond the PZC thus reduce the initial driving force for precipitation and, once formed, promote the redissolution of amorphous iron (hydr)oxide intermediates such as 2-line ferrihydrite.

As the formation of such amorphous or microcrystalline phases generally precedes the stabilization of their thermodynamically more favorable counterparts,^37^ the apparent reduction in thermodynamic driving force and the observed increase in the rate of redissolution compete with one another. Despite an abundance of literature on the transformation of 2-line ferrihydrite, a detailed investigation into the underlying kinetic mechanism remains challenging for a number of reasons. First, most studies are concerned with the transformation of redispersed 2-line ferrihydrite and do not consider the competition between primary nucleation of 2-line ferrihydrite and its redissolution.^20,23,29^ Second, solids are often in the presence of highly concentrated impurities that are readily incorporated into amorphous iron-bearing phases, further delaying phase transformation.^38,39^ Last, the majority of studies reviewed solely quantify the transformation of 2-line ferrihydrite into hematite/goethite in terms of their solid fractions. As the ability of these iron (hydr)oxides to scavenge As, Sr, Cd, and other EOC from highly alkaline radioactive waste and air pollution control residues depends on the ionic strength, it is equally important to also measure the simultaneous turnover of the aqueous Fe(III) concentration coinciding with the solid-phase transformation.

This study investigates the transformation of 2-line ferrihydrite from supersaturation at highly alkaline pH ≥ 13 via a combination of time-resolved X-ray absorption spectroscopy (XAS), thermogravimetric analysis (TGA), X-ray diffraction (XRD), and inductively coupled plasma optical emission spectroscopy (ICP-OES) studies. The resulting data were used to establish a relationship between the rate of 2-line ferrihydrite transformation and the OH^–^ activity across a broad range of conditions reaching from acidic (pH = 2) to highly alkaline (pH = 14) media. The results obtained will help to model the long-term performance of radioactive waste repositories and give further insights into the formation of corrosion products as well as the stability and sorption capacity of 2-line ferrihydrite in alkaline environments including the pore solution of cementitious systems and radioactive waste tailings.

## Materials and Methods

### Synthesis of Pure Iron (Hydr)oxide Phases

The synthesis of all (hydr)oxide phases follows the recommendations of Schwertmann and Cornell.^1^ To enhance product crystallinity above the levels obtained by an Fe(III) iron source and eliminate the system favoring the formation of α-FeOOH(s), γ-FeOOH(s) was synthesized from FeCl_2_·4H_2_O(cr) (Sigma-Aldrich, ReagentPlus, 98%, CAS: 13478-10-9) at room temperature. 60 mmol of FeCl_2_·4H_2_O(cr) was added to 300 mL of UPW (18.2 MΩ cm) under rigorous stirring. Upon bubbling the starting solution with air at a flow rate of ∼120 mL min^–1^, lepidocrocite is formed according to
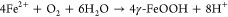
1

To neutralize the protons released during the hydrolysis and maintain the solution at a pH of 6.8 ± 0.1, a total of 130 mL of 1 M NaOH was added incrementally. The first 100 mL was added dropwise through a burette, while the remainder was pipetted at volumes of 250 μL at a time to enable better pH control. The reaction ran to completion after ∼2.5 h, as indicated by a constant pH as well as the characteristic color change from dark blue-greenish to first gray and then orange-yellow. The product was centrifuged and dried in an oven at 40 °C for 2 days. 2-Line ferrihydrite was synthesized by dissolving 100 mmol of Fe(NO_3_)_3_·9H_2_O(cr) (Sigma-Aldrich, ACS reagent grade, ≥98%, CAS: 7782-61-8) in 500 mL of UPW. Throughout the course of the reaction, 350 mL of 1 M KOH was added to neutralize the protons released during Fe^3+^ hydrolysis according to
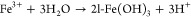
2

The pH was maintained at 7.5 ± 0.1 by adding the last 20 mL of KOH, 500 μL at a time. Amorphous ferrihydrite-containing precipitates are prone to further phase modification, even if stored at dry powders or immersed in water at ambient temperature.^20^ For this reason, the product was not oven-dried, instead, centrifuged at 10,000 rpm for 15 min, and subsequently freeze-dried for 2 days. The identity of the synthesized [2l-Fe(OH)_3_(s) and γ-FeOOH(s)] and purchased [α-FeOOH(s), α-Fe_2_O_3_(s), and α-Fe_3_O_4_(s), Thermo Fisher Scientific, Waltham, MA, USA] iron (hydr)oxide reference standards was confirmed by XRD (Supporting Information, Figure S5).

### Precipitation Experiments

Supersaturated iron stock solutions were prepared by pipetting 5 mL of 1 M FeCl_3_·6H_2_O(cr) in 2 wt % reagent-grade HNO_3_ at pH 1.0 into 245 mL of 0.104, 0.325, and 1.024 M NaOH to yield a final pH of 13.0, 13.5, and 14.0, respectively. The initial concentration of dissolved iron was thus constant at 20 mM across all of the experiments conducted. Stock solutions were aged at ambient temperature in fresh polyethylene containers that were rinsed repeatedly with UPW (18.2 MΩ cm) before use and stirred continuously throughout the duration of the experiment. The type, shape, and crystallinity of iron oxides are heavily influenced by their local chemical environment and the presence of other aqueous species that may be incorporated into the phase.^40,41^ To assess the dependence of their phase assemblage on the iron source utilized, selected stock solutions were prepared from 1 M Fe(NO_3_)_3_·9H_2_O(cr) at the same degree of supersaturation.

Analogous to the postsynthesis treatment of 2-line ferrihydrite, solid precipitates were extracted from stock solutions by centrifugation and subsequent freeze-drying. To avoid a phase transition after centrifugation, the solids were not washed. They were stored as dry powders, and their phase compositions were analyzed after 20 min to 30 days by means of XAS, TGA, and XRD. Here, each point in time corresponds to a freshly prepared stock solution. Aqueous iron concentrations were investigated by ICP-OES throughout the first 2 h of the experiment. The concentration at each point in time corresponds to the arithmetic mean of the concentrations extracted from three independently prepared stock solutions. Prior to analysis, aliquots of ∼1 g were taken from aged alkaline solutions, immediately filtered using 0.20 μm nylon filters (Semadeni AG, Ostermundigen, Switzerland), and acidified in 2 wt % HNO_3_ in UPW at a ratio of 1:10 wt % to prevent further precipitation. The matrix was prepared from 65 wt % HNO_3_ (EMSURE), from Merck Group (Merck KGaA, Darmstadt, Germany). No more than 10 aliquots of ∼1 g were taken from the same reservoir.

### Analytical Methods

#### Scanning Electron Microscopy

All scanning electron microscopy (SEM) images were obtained at the ETH Zürich Scientific Center for Optical and Electron Microscopy (ScopeM) using a Hitachi SU5000 at a working distance of 4.8 mm under a high vacuum and 3 kV voltage. The samples were sputter-coated with a Pt/Pd (80/20%) alloy and placed on an aluminum sample holder using carbon tape.

#### Synchrotron-Based Investigations

XAS spectra at the Fe K-edge were collected at PHOENIX I (Photons for the Exploration of Nature by Imaging and XAFS) at the Swiss Light Source (SLS), Paul Scherrer Institute (PSI), Villigen, Switzerland. The beamline allows for measurements in the tender X-ray region, ranging from 0.8 to 8.0 keV. The photon source is a linearly and elliptically polarizable APPLE II undulator, granting a flux of ∼1 × 10^12^ photons s^–1^ at 3 keV.

Measurements were conducted under vacuum (∼1 × 10^–6^ bar) and ambient temperature, employing two detection modes simultaneously: (i) the total electron yield (TEY) and (ii) the total fluorescent yield (TFY) at a beam size of 0.9 × 0.9 mm^2^. The sample TFY was recorded with a four-element vortex detector. The incident flux, *I*_0_, was measured as TEY signal taken from a Ni-coated polyester foil. The polyester foil was mounted on an electrically insulated holder, which was located in a separate chamber at ∼5 × 10^–8^ bar, located 1 m away from the sample. The monochromator [Si(111)] was calibrated by assigning the first inflection point of a reference iron foil to 7111.08 eV.

Iron oxide samples were uniformly applied to conductive carbon tape and placed on a copper sample holder that had previously been roughened by sand paper to ensure a noise-free total electron signal. Reference compounds and powders extracted during precipitation experiments were measured 3–5 times and their spectra were normalized and averaged using the Athena interface of the IFFEFIT software package.^42,43^ No attempt has been made to smooth the data beyond glitch removal. Solid spectra were corrected for self-absorption by comparing and adjusting the fluorescent signal to that of the TEY. Extended X-ray absorption fine edge structure (EXAFS) data were measured with a duration of ∼24 min per scan, converted into *k*^3^-weighted χ(κ), and subsequently Fourier-transformed into the *R*-space with the Kaiser–Bessel window function between 1.5 and 10.0 Å^–1^. Time-resolved XAS spectra of iron (hydr)oxide precipitates were fitted to reference compounds by linear combination fitting (LCF) in *k* space over 2.0–9.0 Å^–1^, forcing the sum of all weightings to add up to 1. A maximum of five reference standard spectra, those of 2l-Fe(OH)_3_(s), α-FeOOH(s), γ-FeOOH(s), α-Fe_2_O_3_(s), and α-Fe_3_O_4_(s), and all combinations in-between, were considered. The identity and purity of reference standards utilized were confirmed by XRD and TGA.

#### Thermogravimetric Analysis

Substances were analyzed in a Netzsch STA 449 F3 Jupiter. All samples were heated from 30 to 1000 °C at 10 °C min^–1^ under a nitrogen atmosphere and show an initial mass of 30 ± 0.1 mg. The nitrogen gas purge flow rate was 20 mL min^–1^, and the alumina crucible mass was 325 ± 5.0 mg. To quantify the conversion of one iron oxide to another, their respective derivative thermogravimetry (DTG) curves were integrated via the tangential method^44^ (Supporting Information, Figure S2).

#### X-ray Diffraction

XRD measurements were performed on powders extracted from iron stock solutions. Diffraction patterns were obtained using a Bruker D8 Advance diffractometer with automatic beam optimization in a coupled 2θ–θ configuration using Co Kα radiation (λ = 1.7902 Å) and a LynxEye XE-T detector. Samples were measured between 4 and 80° in steps of 0.02 2θ, and their patterns were analyzed and compared to reference powder diffraction files of 2-line ferrihydrite,^45^ lepidocrocite (PDF entry 00-044-1415), goethite,^46,47^ hematite (PDF entry 00-033-0664), and magnetite (PDF entry 00-019-0629) using the open-source XRD and Rietveld refinement program Profex.^48^ Sizes of the coherently scattering crystal domain were estimated by applying the Scherrer equation.^49^ It is assumed that their standard deviation was approximately equal to 10% of the domain size.^22,50^

#### Inductively Coupled Plasma Optical Emission Spectroscopy

Analyses were carried out using an Agilent 5110 ICP-OES instrument (Agilent Technologies Inc., Santa Clara, CA, USA), equipped with an Agilent SPS 4 autosampler. The total amount of dissolved iron was correlated with the recorded intensity via an 8-point calibration line in-between the range of 0.01–50.00 mg L^–1^ (Supporting Information, Figure S3). The computed limit of detection (LOD) and limit of quantitation (LOQ) as well as the elemental composition of all standards are reported in Tables S1 and S2. The aqueous concentration of iron in equilibrium with the solid iron (hydr)oxide phases stabilized is in the order of μg L^–1^ and hence differs from the initial amount of iron dissolved by 3–4 orders of magnitude. Here, particular caution was exercised to determine the accuracy and precision of the analytical method and assess potential measurement interferences due to other high-concentration elements including Na and K present in the order of g L^–1^. To avoid spectral interferences, 4 analyte emission lines, namely, Fe 234.350, 238.204, 239.563, and 259.940 nm, were considered. In line with the recommendations of Caruso et al.,^51^ it was decided to determine the concentrations based on the selected spectral line at 259.940 nm. A more detailed account of the respective LOD and LOQ as well as a comparison of the progression of [Fe] with time as determined via various calibration curves are presented in the Supporting Information.

## Results

### Mineral Phase Identification

**Figure 1:**
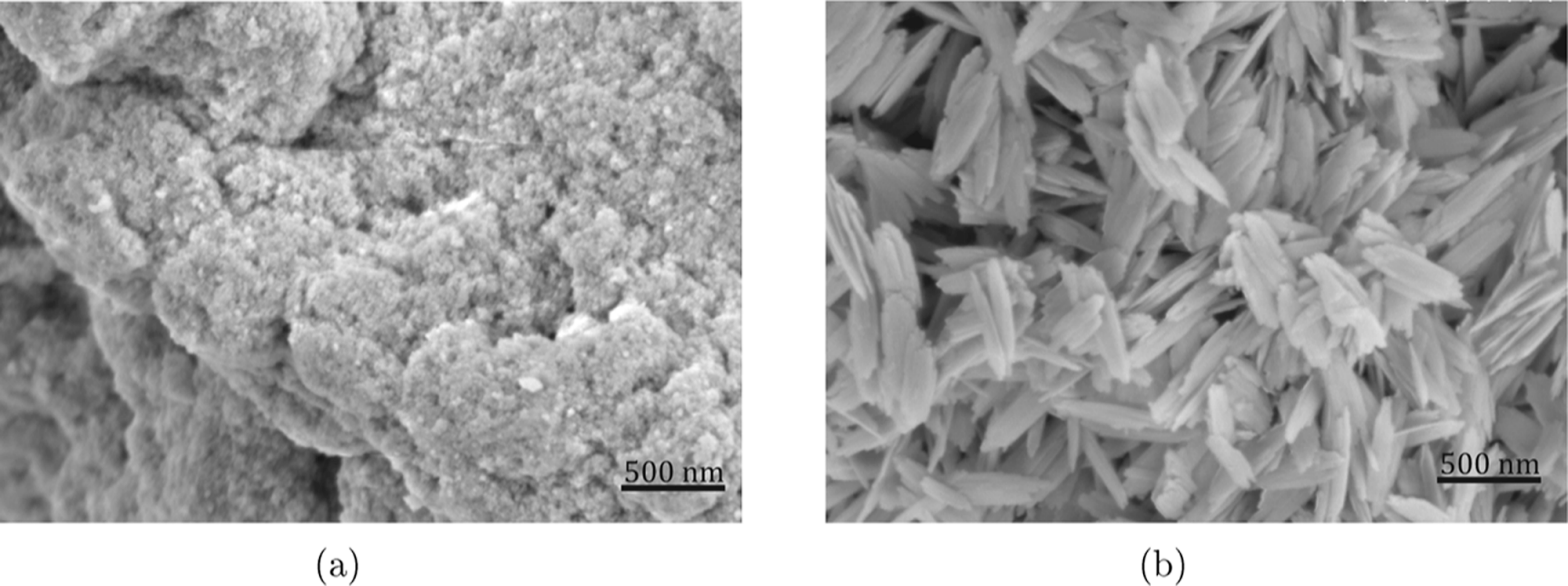
SEM images of iron (hydr)oxide powders extracted from supersaturated stock solutions at an alkaline pH. (a) Amorphous aggregates extracted after 20 min of equilibration time and (b) needle aggregates equilibrated after 30 days.

SEM images of powders extracted from supersaturated iron stock solution clearly show a transition toward more crystalline aggregates. As schematically illustrated in Figure 1, iron (hydr)oxide powders extracted after 20 min of equilibration time appear lath-like and irregular, similar in appearance to aggregations of ferrihydrite nanoparticles.^52^ Within 30 days, the morphology transformed completely into needlelike crystals typical for the iron hydroxide goethite. Moreover, both samples are free of platelet crystals characteristic to the morphology of lepidocrocite.^53^

**Figure 2:**
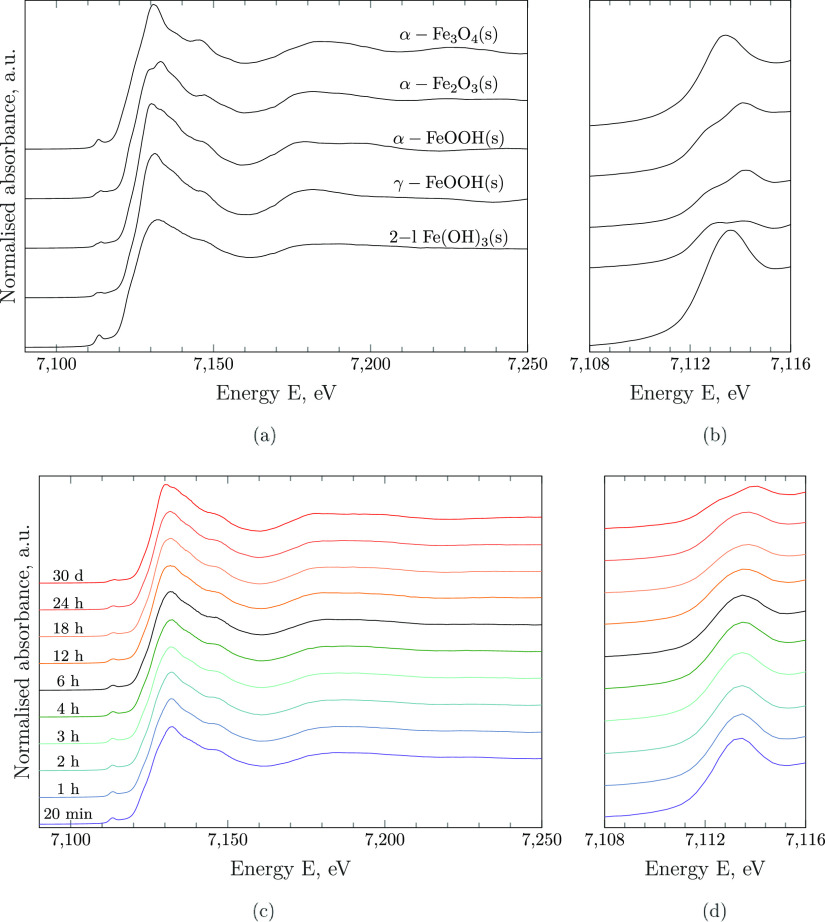
Normalized Fe K-edge XANES and pre-edge spectra of iron (hydr)oxide reference components (a,b) and solids extracted from supersaturated iron stock solutions (c,d) containing 20 mM FeCl_3_·6H_2_O(cr) at pH = 14.0. All spectra displayed correspond to the self-absorption-corrected fluorescent signal.

The average oxidation state and coordination of crystalline iron-bearing phases can be determined by the position and intensity of their characteristic XAS pre-edge located approximately 15–20 eV before the main K-edge.^54,55^ While the average redox state largely depends on the centroid position of the pre-edge feature, integrated peak intensities, among other reasons, are determined by the degree of centrosymmetry.^56^ Among the 6-fold oxygen-coordinated Fe^3+^-bearing reference components as presented in Figure 2, 2-line ferrihydrite shows the highest integrated peak intensity, followed by lepidocrocite, goethite, and hematite. This trend is to be expected, as peak intensities scale with the degree of octahedral Fe(O,OH)6 polymerization.^54^ Fe K-edge XANES spectra of iron (hydr)oxide solids extracted from supersaturated stock solutions at pH = 14.0 (Figure 2c,d) demonstrate a clear transition toward more centrosymmetrically coordinated Fe^3+^ bearing minerals, i.e., from 2-line ferrihydrite toward a more crystalline iron hydroxide. As evident from Figure 2d, the pre-edge feature gradually reduces in intensity. Moreover, the relative contributions of the 1s → 3d/4p^56,57^ transitions shift over time, leading first to broadening and then to splitting of the pre-edge peak. In the absence of extra transitions at ∼7114 eV related to Fe clustering as visible in the reference spectrum of hematite,^58^ the peak splitting observed is characteristic to variants of the FeOOH(s) phase such as lepidocrocite and goethite.

**Figure 3:**
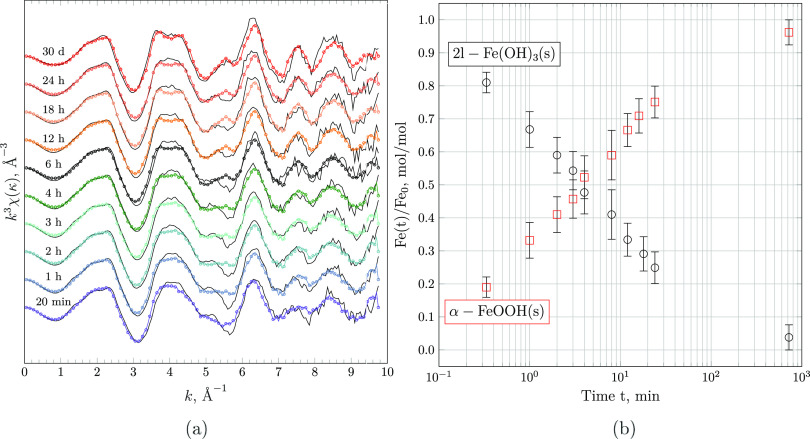
Fe K-edge EXAFS *k*^3^χ(κ) (solid black lines) linear combination fits (colored marker lines) of aged solid samples from a supersaturated FeCl_3_·6H_2_O(cr) stock solution at pH = 14 (a) and time-dependent fractions of the respective reference solids obtained from LFC (b). Fits were achieved using the reference standards 2l-Fe(OH)_3_(s) and α-FeOOH(s), and the fitting range was 2–9 Å^–1^.

To quantify the extent of phase transformation, the Fe K-edge XAS spectra of iron (hydr)oxide solids have been transformed into the *k*-space and subsequently fitted to all combinations within the pool of reference spectra (Supporting Information, Figure S6). At all equilibration times, the best fits have been universally achieved by solely including the spectra of 2-line ferrihydrite and goethite. In detail, the inclusion of lepidocrocite as a reference compound did not improve the fitting and predicted close to zero value molar fractions within the fitting error throughout (Supporting Information, Figure S7). Fe K-edge EXAFS *k*^3^χ(κ) fitting results and the respective mole fraction of 2-line ferrihydrite remaining and goethite formed are displayed in Figure 3. It must be emphasized that, even though both standards show distinct EXAFS spectra, linear combination fitting may underestimate the amount of goethite present, depending on the crystallinity and particle size.^59^

**Figure 4:**
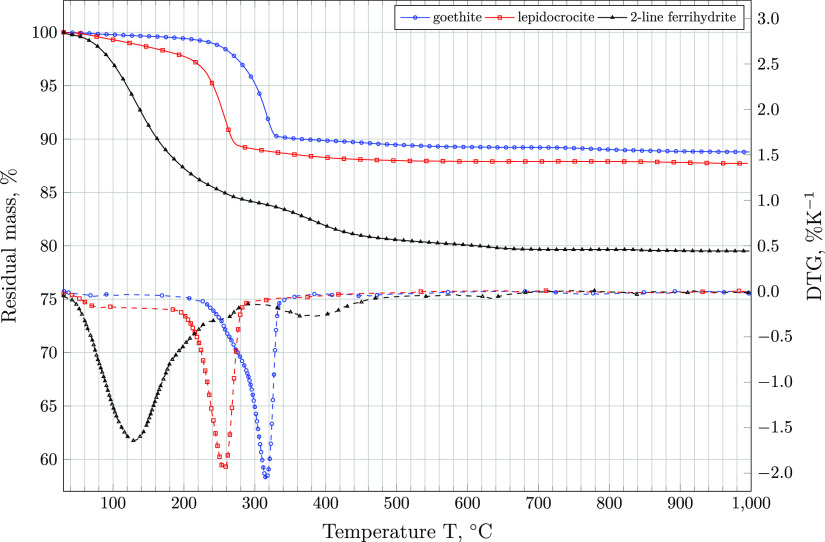
Percentage residual mass (continuous) and DTG (dashed) curves of reference components 2-line ferrihydrite, lepidocrocite, and goethite.

**Figure 5:**
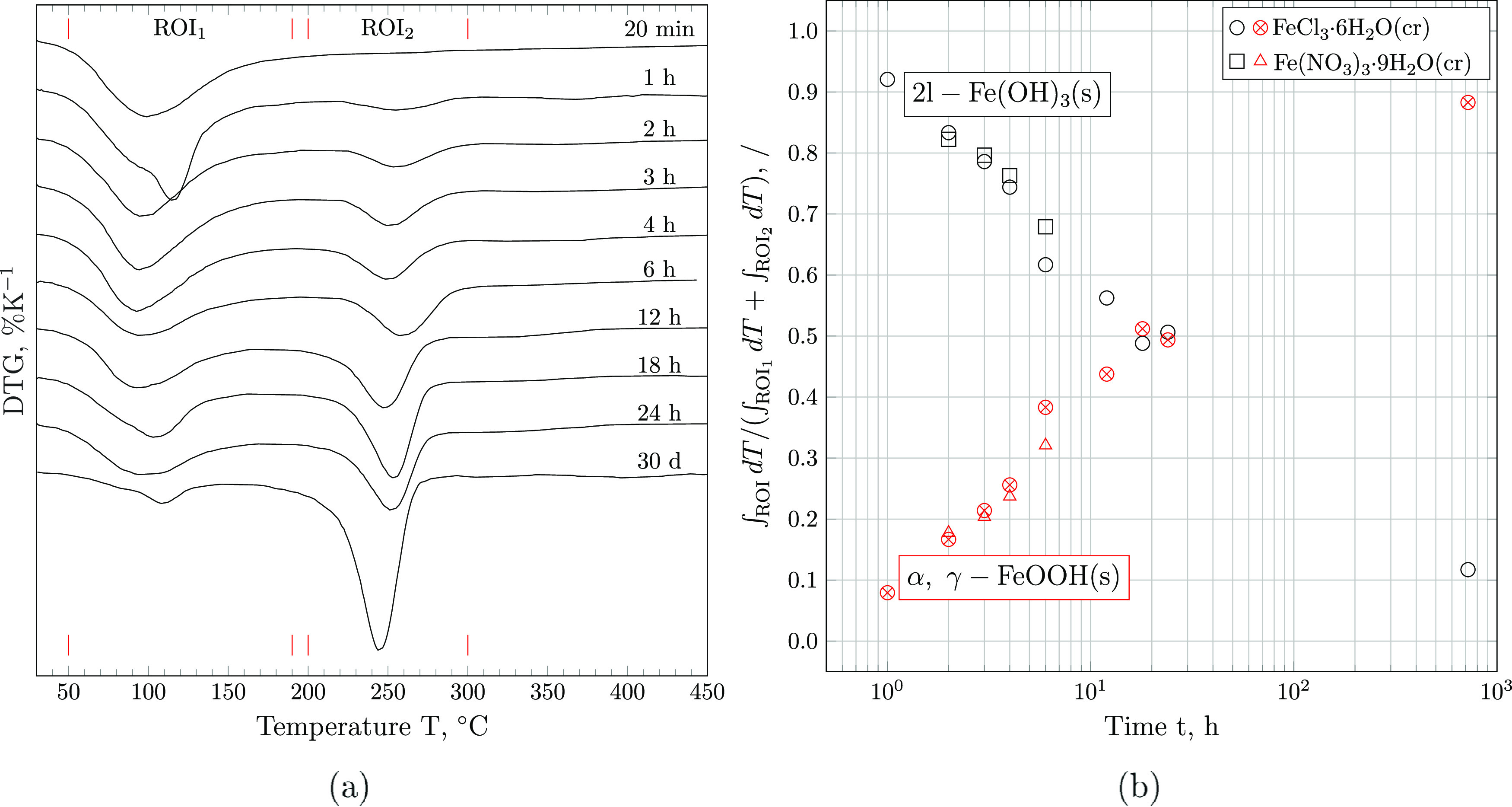
Progression of DTG curves of iron oxide samples extracted from 1 M NaOH, pH = 14.0, at different equilibration times. Solids formed from 20 mM FeCl_3_·6H_2_O(cr) are marked with a circle, while those stabilized from 20 mM Fe(NO_3_)_3_·9H_2_O(cr) are denoted with square markers. (a) DTG curves of all FeCl_3_·6H_2_O(cr) samples together with the ROI for both characteristic peaks observed. (b) Area underneath each ROI, relative to the total weight loss over both peaks. All areas have been evaluated via the tangential method,^44^ integrated over the temperature interval [50, 190] and [200, 300], respectively.

Phase changes determined via Fe K-edge EXAFS linear combination fitting are further augmented by TGA and XRD of the very same iron (hydr)oxide powders extracted. Comparison between the DTG curves of pure reference components (Figure 4), other synthetic and naturally occurring iron (hydr)oxide samples,^38,60−64^ and those of the samples investigated in this study (Figure 5) suggests the stabilization of goethite or lepidocrocite from 2-line ferrihydrite.

As shown in Figure 4, the reference 2-line ferrihydrite shows a weight loss of 3.0 and 17.5% before and exceeding 100 °C, respectively. Discrepancies between the theoretical water content of ∼17% and the total weight loss recorded can be attributed to varying extents of physisorbed water,^38^ arising from to differences in sample synthesis and post-treatment.^38,60,61^ The main weight loss occurs at temperatures lower than 250 °C, indicating that 2-line ferrihydrite contains very little structural OH.^65^ In contrast, the evaporation of free water from lepidocrocite and goethite occurs at temperatures <100 °C.^62^ Major weight losses of lepidocrocite due to its dehydration to hematite occur at 200 ≤ *T* ≤ 275 °C.^62^ Goethite reference samples investigated loose the majority of their weight due to the evaporation of chemically bound water and the associated phase transformation to hematite in the same temperature interval, though the exact DTG peak position differs from that of lepidocrocite by ∼50 °C. The exact temperature at which FeOOH(s) losses water as well as the residual weight depends on the amount of FeOOH present in the TGA as well as its crystallite and particle size.^44,63^

The DTG curves of iron (hydr)oxides extracted at different equilibration times show two distinct peaks, (i) at *T* ∼ 100 °C and (ii) 200 ≤ *T* ≤ 300 °C, matching the temperature intervals across which (i) 2-line ferrihydrite and (ii) lepidocrocite and goethite lose the majority of their weight. As illustrated in Figure 5, the weight loss across the first region of interest (ROI), i.e., from 50 to 190 °C, relative to the area underneath both peaks decreases exponentially with time. Conversely, the fraction of weight loss occurring across the temperature interval from 200 to 300 °C increases by the same amount, as shown in Figure 5b. Assuming that water losses within the first ROI (50–190 °C) are solely due to the decomposition of 2-line ferrihydrite, the amount of 2-line Fe(OH)_3_(s) may be computed according to
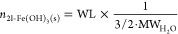
3where WL is the observed weight loss as computed via the tangential method^44^ and  is the molecular weight of water in g mol^–1^. The dehydration of α-FeOOH(s) (goethite) and γ-FeOOH(s) (lepidcrocite) to 1/2α-Fe_2_O_3_(s) (hematite) in the second ROI (200–300 °C) can be quantified according to
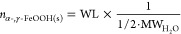
4

**Figure 6:**
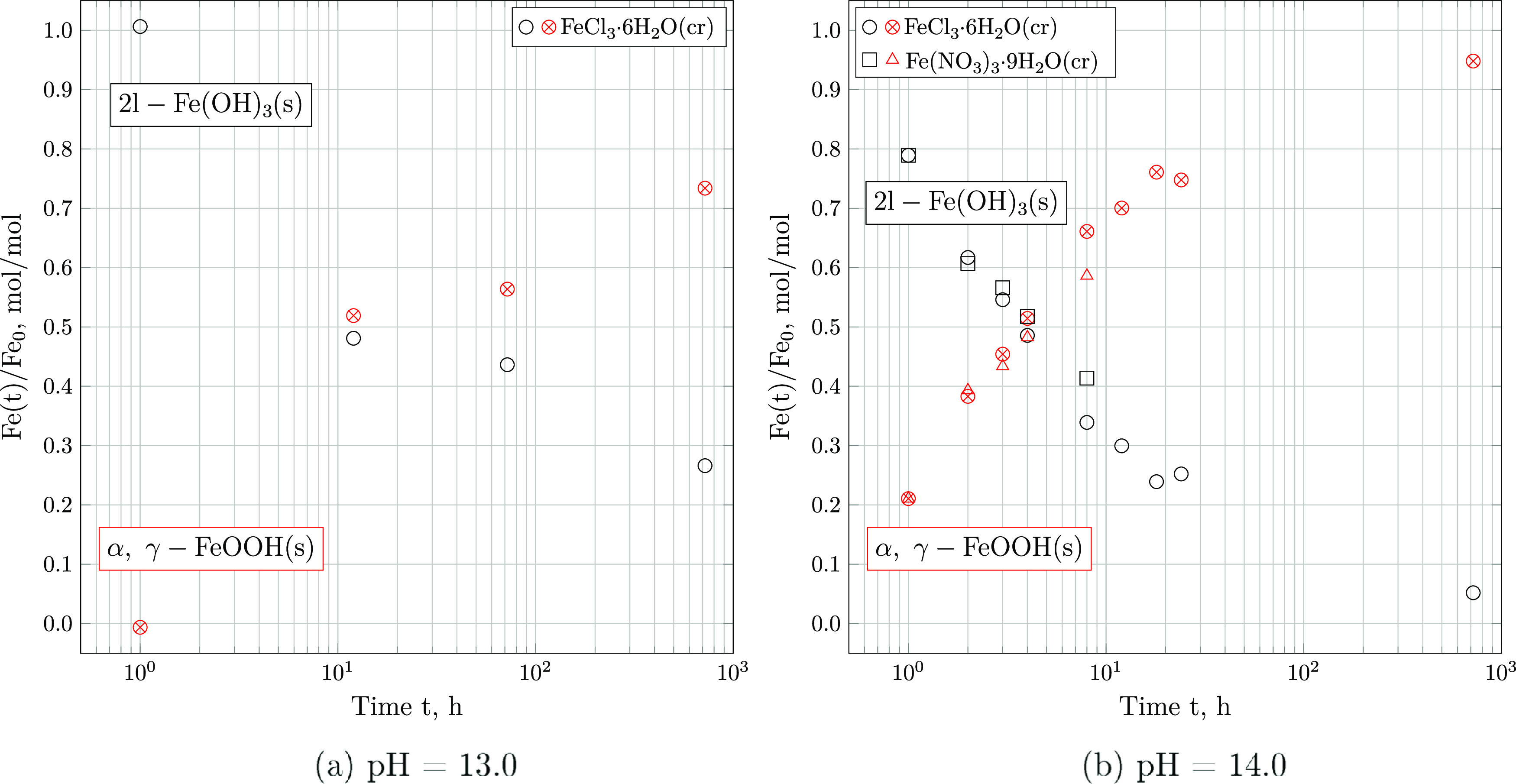
Progression of the mass fraction of 2l-Fe(OH)_3_ and FeOOH(s)-type iron hydroxides stabilized at pH = 13.0 (a) and pH = 14.0 (b) over time, as determined by TGA and computed by eq 4. Solids formed from FeCl_3_·6H_2_O(cr) are marked with a circle, while those stabilized from Fe(NO_3_)_3_·9H_2_O(cr) are denoted with square markers.

As evident from Figure 6, mass fractions computed directly from the weight losses of water on the respective temperature intervals differ by about 10% compared to mole fractions inferred by eqs 3 and 4. It is further evident that the conversion proceeds significantly more rapidly at pH = 14.0. Moreover, the reaction coordinate appears to be independent of the iron source used, as 2l-Fe(OH)_3_ stabilized from both FeCl_3_·6H_2_O(cr) and Fe(NO_3_)_3_·9H_2_O(cr) converts to FeOOH(s) at the same rate.

In addition to the evaporation of physisorbed water, samples prepared from Fe(NO_3_)_3_·9H_2_O(cr) may lose NO_2_(g) if present in the solid fraction at ∼150 °C, i.e., within the first ROI (50–190 °C) and samples synthesized from FeCl_3_·6H_2_O(cr) eliminate any Cl_2_(g) associated with the solid phase at ∼190–210 °C,^66^ overlapping with the second ROI. As for the Cl containing sample, no significant weight loss was observed at 200 °C after 20 min (i.e., before any goethite had formed), and no significant amount of Cl_2_(g) seems to have been associated with solid phase. As the respective molar fractions of 2-line ferrihydrite and goethite agree well across both iron sources used, it can be concluded that weight losses due to NO_2_(g) and Cl_2_(g) do not contribute significantly to the overall conversion rate calculated. An unambiguous characterization of the FeOOH(s) phase is not possible via TGA, as the second ill-defined DTG peak is located within the overlapping region of both higher stability iron hydroxides.

**Figure 7:**
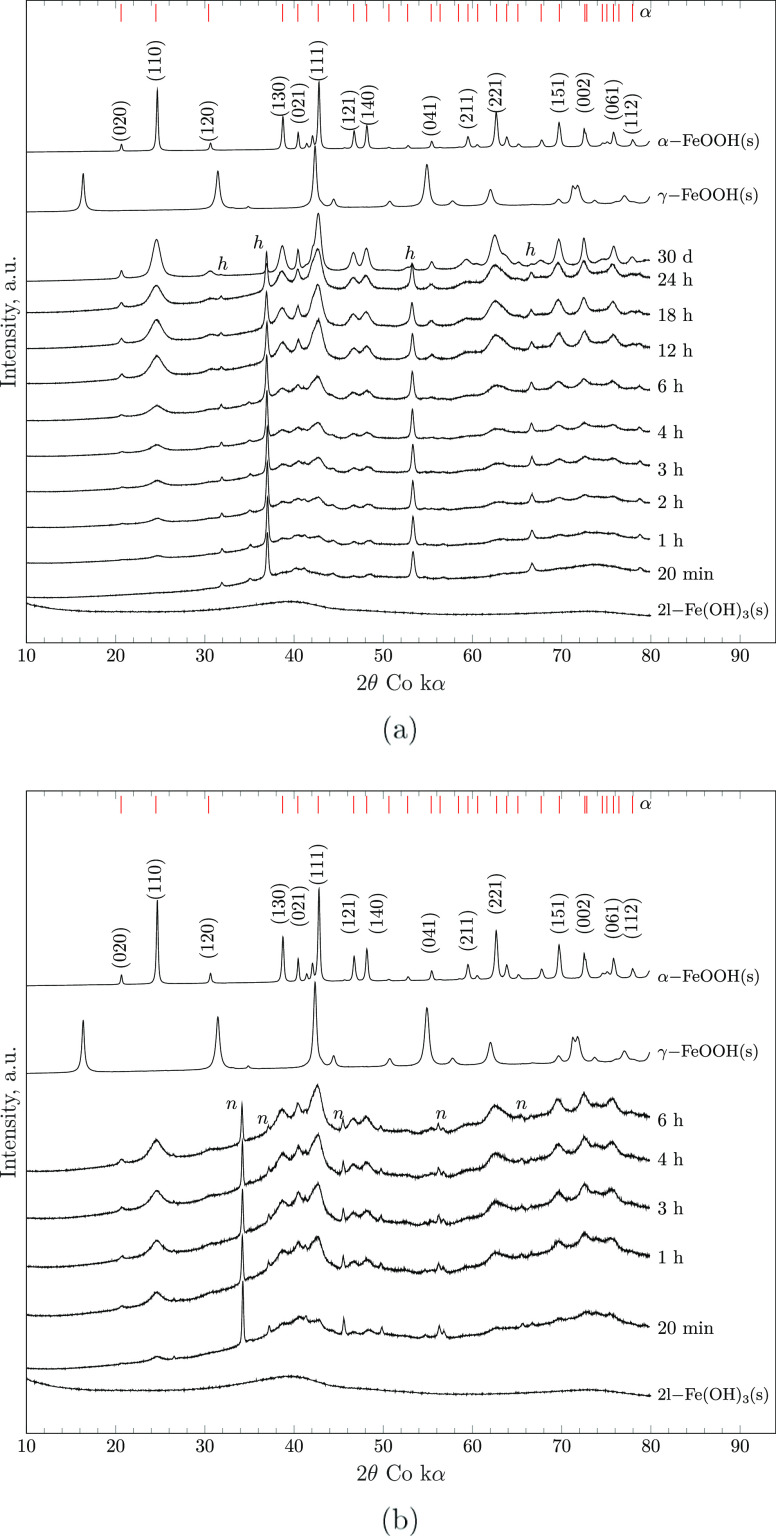
XRD patterns of time-dependent iron (hydr)oxide phases formed from 20 mM FeCl_3_·6H_2_O(cr) (a) and 20 mM Fe(NO_3_)_3_·9H_2_O(cr) (b) in 1 M NaOH (pH 14), together with the patterns of reference compounds 2l-Fe(OH)_3_(s), α-FeOOH(s), and γ-FeOOH(s). Position of the main peaks of goethite (α-FeOOH(s)) is marked as red —, while the peaks of halite (NaCl) and sodium nitrate (NaNO_3_) are denoted by h and n.

X-ray diffractograms further demonstrate that the FeOOH(s)-type iron hydroxide stabilized from 2-line ferrihydrite is goethite (α-FeOOH(s)). As displayed in Figure 7, the solids extracted after 20 min from supersaturated iron stock solutions feature two broad peaks centered at 2θ of ∼40 and ∼74°, similar to those of the reference powder diffraction file^45^ and the synthesized 2-line ferrihydrite sample (Supporting Information, Figure S5). Over time, the characteristic 110 and 111 peaks of goethite at 2θ of ∼24 and ∼42° emerge from the amorphous diffractograms initially recorded. It is also evident that both 2-line ferrihydrite and goethite are formed within the first few hours of equilibration. Subsequently, the crystallinity of the final product goethite increases, and no other (iron-bearing) phase is formed. This trend holds true, irrespective of the iron source used during precipitation experiments. Both the time series for batches using FeCl_3_·6H_2_O(cr) and Fe(NO_3_)_3_·9H_2_O(cr) show peaks of their respective residual crystalline side products halite and sodium nitrate, as marked by the letters h and n.

**Figure 8:**
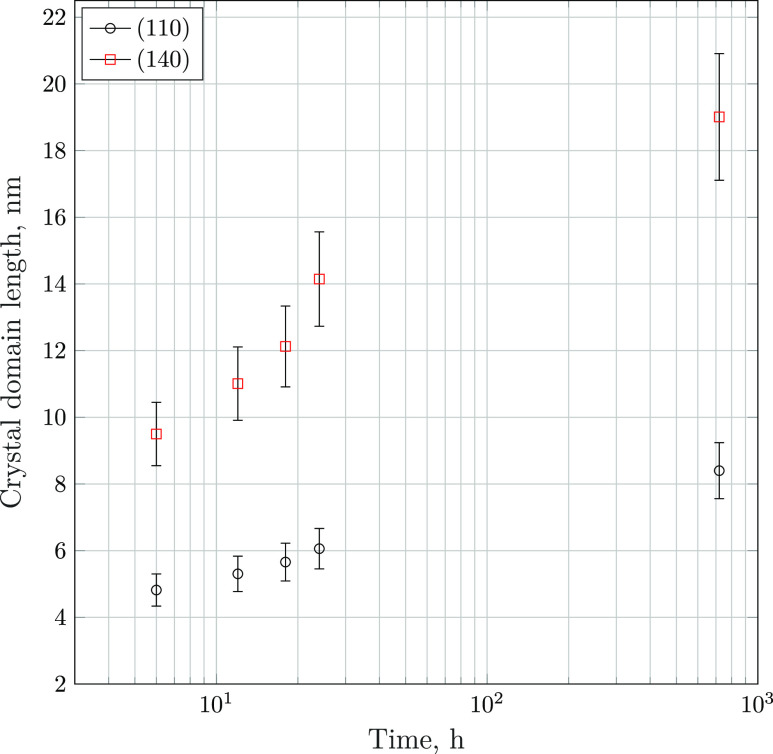
Size of the coherently scattering crystal domain of selected goethite (α-FeOOH(s)) peaks at pH = 14, as estimated via peak shape analysis and the application of the Scherrer equation.^49^

The incident transformation of the crystal structure to the orthorhombic lattice of goethite is concluded within 30 days. XRD peak analysis further confirms that the size of the coherently scattering crystal domain of goethite grows continuously over the timespan investigated. Figure 8 illustrates the increase in domain size along the 110 and 140 directions as a function of aging time.

Even though the combined analysis of X-ray diffractograms in conjugation with the computed molar fractions of iron (hydr)oxides, as determined by EXAFS LCF and DTG, suggests the formation of α-FeOOH(s) solely from 2l-Fe(OH)_3_(s), the presence of lepidocrocite or 6-line ferrihydrite as an intermediate phase or in minor quantities below the XRD detection limit or within the EXAFS fitting error cannot be completely excluded. The presence of lepidocrocite as transitional phase in quantities below the detection limit of both techniques may be further investigated using microscopic techniques.^33,53^ As illustrated in Figure 1, the SEM images of iron (hydr)oxide phases recorded show no evidence of the characteristic platelet lepidocrocite crystals, further supporting the proposed phase transformation of 2-line ferrihydrite to goethite.

### Quantification of the Total Aqueous Iron Concentration

**Figure 9:**
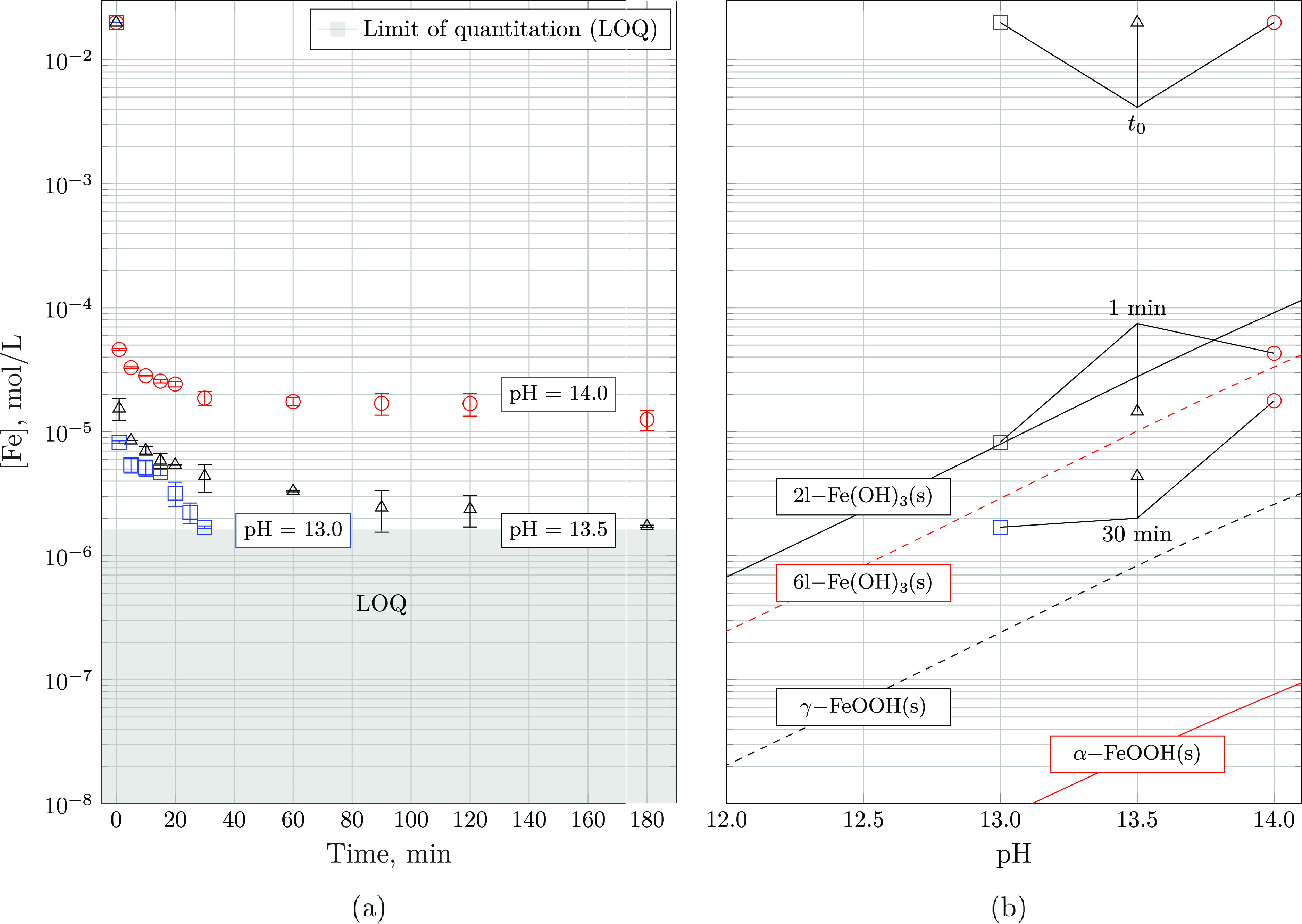
Total iron concentration as measured by ICP-OES. (a) Progression of [Fe] in mol L^–1^ over time for pH 13.0, 13.5, and 14.0 at ambient temperature. Error bars represent the standard deviation in the measurements of three independently prepared solutions. (b) Concentration at selected points in time versus the solubility of Fe(III), as controlled by the solubility of 2- and 6-line ferrihydrite (2-, 6-l Fe(OH)_3_(s)), lepidocrocite (γ-FeOOH(s)), and goethite (α-FeOOH(s)) taken from Furcas et al.^36^

The total iron concentration as a function of time and pH as determined by ICP-OES is displayed in Figure 9. Irrespective of the pH, iron concentrations decrease from 20 mM to the order of tens of μmol L^–1^ within the first minute. Across this timespan, the initial precipitation rates are estimated to be (3.331 ± 0.004) × 10^–4^ mol L^–1^ s^–1^, irrespective of the pH. After this rapid initial decrease, the progression of [Fe] flattens out, striving toward some finite, pH-dependent solubility limit. While iron concentrations at pH = 14.0 remain on the order of 10^–5^ M, those at pH = 13.0 are below the limit of quantification after 30 min. This is expected as the concentration of Fe(III) decreases and the degree of supersaturation with respect to any of the iron (hydr)oxides increases with decreasing pH.

Concentration measurements are generally consistent across all precipitation experiments (Figure 9) and spectral lines (Figure S4) at each time step. After 1 min and longer, the measured iron concentrations were at or below the solubility of 2-line ferrihydrite. After 30 min, all concentrations were below the solubility of 6-line ferrihydrite, which was not observed to form by either XAS or XRD. As time progresses, concentrations align parallel to the solubility limits of various iron (hydr)oxides, and the system remains supersaturated with respect to lepidocrocite, goethite, and other high-stability phases including hematite and magnetite. This implies that at least one of the iron (hydr)oxides formed features a solubility lower than that of 6-line ferrihydrite. The time-resolved experimental techniques employed here demonstrate that the formation of stable iron (hydr)oxide phases at alkaline pH comprises the initial rapid precipitation of amorphous 2-line ferrihydrite as the intermediate phase, followed by its transformation to goethite. The continual decrease of the aqueous iron concentration coinciding with phase transformation over time suggests that the precipitation of goethite likely occurs from solution. On the other hand, the rapid decrease of the molar fraction of 2-line ferrihydrite in equilibrium with the aqueous phase indicates that other mechanisms including oriented attachment (OA) may take place simultaneously. The formation of 6-line ferrihydrite and lepidocrocite, which were both oversaturated during the experiments, was not observed, indicating that their formation kinetics are slower at pH 13.0–14.0.

### Kinetics of 2-Line Ferrihydrite Transformation

To compare the transformation rates measured here at highly alkaline pH to those measured in acidic and circumneutral environments, time-dependent concentration profiles of 2-line ferrihydrite obtained by EXAFS linear combination fitting and those measured by Schwertmann et al.,^23^ Schwertmann and Murad,^24^ and Das et al.^29^ were plotted together. The rate constants were calculated by the integrated first-order rate equation
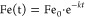
5

**Figure 10:**
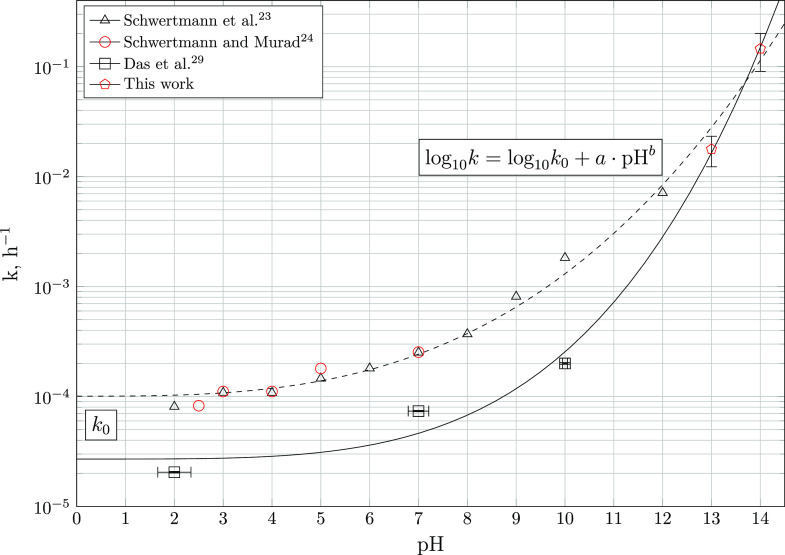
Estimated first-order rate constants of 2-line ferrihydrite transformation calculated by Das et al.^29^ and obtained by fitting eq 5 to the progression of the molar fraction of 2-line ferrihydrite at 23 ± 2 °C, as determined by EXAFS LCF and TGA and measured by Schwertmann et al.^23^ and Schwertmann and Murad.^24^ The rate constant obtained in this work at pH = 14.0 represents the average rate constant and error achieved by fitting the estimated mole fractions from EXAFS LFC and TGA, while the rate constant at pH = 13.0 is derived from TGA only, assuming the same uncertainty. Plots of the fits achieved are shown in Figures S8 and S9.

Figure 10 shows the calculated first-order rate constant *k* in h^–1^ as a function of the pH. It can be recognized that the rate of transformation of 2-line ferrihydrite to goethite (and hematite at 5 ≤ pH ≤ 10) strictly increases as a function of the activity of OH^–^ or pH. In acidic to neutral conditions, the rate constants derived based on the data of Schwertmann and co-workers^23,24^ vary by less than 1 order of magnitude, reaching values of 8.1 × 10^–5^ h^–1^ at pH = 2.0 and 2.5 × 10^–4^ h^–1^ at pH = 7.0, respectively.

Various *k* calculated by Das et al.^29^ are slower than those calculated by re-evaluating the data of Schwertmann et al.^23^ by up to 1 order of magnitude. While Schwertmann et al.^23^ used the fraction of oxalate-soluble iron hydroxide as an estimate of the degree of transformation, Das et al.^29^ computed the relative amounts of 2-line ferrihydrite remaining via XANES linear combination fitting. As the difference between the total and oxalate-soluble iron hydroxide varies from the amount of goethite determined by XRD and TGA by approximately 5–15%,^67,68^ it is evident that the discrepancy in estimated rate constants are most likely a consequence of the quantification methods employed. Moreover, differences in experimental protocols (e.g., the drying procedure of aliquots) may have contributed to the differences in obtained rate constants. At pH = 14, the here estimated first-order rate constant surpasses that at pH = 2–4 by more than 3 orders of magnitude, reaching a value of 1.5 × 10^–1^ h^–1^. This drastic increase in phase transformation rates across the pH range investigated is well captured by
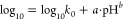
6where *k*_0_ is the standard rate constant at pH = 0 in h^–1^ and *a* and *b* are empirical parameters at 25 °C. Fitting eq 6 to the set of estimated first-order rate constants at disposal results in cubic (*k*_0_ = 1.01 × 10^–4^ h^–1^, *a* = 1.10 × 10^–3^, and *b* = 3 for the data of Schwertmann et al.^23^ and Schwertmann and Murad^24^) or quartic (*k*_0_ = 2.70 × 10^–5^ h^–1^, *a* = 9.74 × 10^–5^, and *b* = 4 for the rate constants calculated by Das et al.^29^) dependence of log_10_*k* on the pH. Considering the apparent discrepancy between the fraction of goethite predicted by selective dissolution and other analytical techniques, it is recommended to use the latter fit, employing rate constants consistently predicted by XAS LCF.^29^

It must be emphasized that the analysis presented in this section does not permit to draw any conclusion regarding the rate of 2-line ferrihydrite formation or the growth mechanism of goethite. Instead, we provide a semiempirical relationship describing the overall transformation rate as a function of the pH, and therefore, the OH^–^ activity. As evident from the SEM images presented in Figure 1, the mechanism leading to the formation of goethite involves changes in the particle morphology and surface area. Moreover, goethite particle growth is known to be inhibited in the presence of both Si and Cl.^1,2^ To account for the effects of other physiochemical parameters in addition to the solution pH and formulate a crystallization mechanism that describes the growth of both minerals in partial equilibrium with the aqueous phase requires careful construction of all kinetic rate laws involved. A formulation of such a mechanism will be the subject of future work.

### Environmental Implications

Observations demonstrate that, despite the fast dissolution kinetics of 2-line ferrihydrite at high pH values, the aqueous concentration of Fe(III) decreases only slowly, and the solutions remain supersaturated with respect to goethite for a significant time. Due to its very rapid formation and slower dissolution, 2-line ferrihydrite can be considered a point source of Fe(III) that maintains the aqueous phase in a state of disequilibrium. Within the alkaline pore solution of cementitious matrices such as those used in radioactive waste storage, the amount of Fe(III) above the solubility limit of goethite can readily be transported across the pore network or taken up by any other cementitious phase in the system, prospectively impacting their service life and long-term ability to contain hazardous radionuclides.^37,69^ As the transformation from amorphous 2-line ferrihydrite to crystalline goethite coincides with a 10-fold reduction in the specific surface area, also the capacity to immobilize toxic elements such as As, Sr, or Cd is expected to be severely reduced. The estimated 2-line ferrihydrite half-life *t*_1/2_ at pH = 10, i.e., at mildly alkaline conditions characteristic to uranium mine tailings,^4,29^ amounts to approximately 15 days at 25 °C. At pH > 13, i.e., the pH characteristic to radioactive waste tailings in Portland cements, ferrihydrite half-life is approximately 40 and 5 h at pH 14, as present in alkali activated cements. However, the rate of 2-line ferrihydrite transformation also depends on the presence of other multivalent impurities that may impede sorption of primary EOC.^1,20,38,39^ To extend the analysis presented in this work to the conditions prevailing in the pore solution of cementitious systems, the effect of silica and carbonates on the transformation mechanism must be investigated. Likewise, uranium mill raffinates are rich in SO_4_^2–^ that could complex with aqueously dissolved iron or form Fe_2_(SO_4_)_3_, further impeding the formation of goethite.^17^ To derive a semiempirical rate expression representative of the aqueous chemistry of these raffinates, kinetic rate constants must be re-evaluated in sulfate-supersaturated media. A rigorous comparison between these competing phenomena requires a more thorough understanding of both the crystallization process including the initial precipitation of 2-line ferrihydrite, the growth of goethite, and the change in particle morphology.

## Supporting Information Available

Quantification of iron hydroxide phases by TGA; calibration of ICP-OES, XRD and XAS fitting, and reference spectra; and first-order fitting results (PDF)
