# Supplementary material for: Transformation of 2-Line Ferrihydrite to Goethite at Alkaline pH
Source: Environ Sci Technol. 2023 Oct 12;57(42):16097–108. doi: 10.1021/acs.est.3c05260 (PMC10603785; doi:10.1021/acs.est.3c05260)
Supplement: Supplementary file 1 — es3c05260_si_001.pdf [file es3c05260_si_001.pdf]

Supporting Information
Transformation of 2-line ferrihydrite to goethite at alkaline pH

Fabio E. Furcas,† Barbara Lothenbach,‡ Shishir Mundra,† Camelia N. Borca,¶ Cristhiana Carine Albert,† O. Burkan Isgor,§ Thomas Huthwelker,¶ and Ueli M. Angst*,†

† *Institute for Building Materials, ETH Zürich, 8093, Zürich, Switzerland*

‡*Empa Concrete & Asphalt Labortory, 8600 Dübendorf, Switzerland*

¶*Swiss Light Source, Paul Scherrer Institut, 5232 Villigen, Switzerland*

§*School of Civil and Construction Engineering, Oregon State University, Corvallis, OR, USA*

E-mail: uangst@ethz.ch

Phone: +41446334024

13 pages, 9 figures, 2 tables

List of Figures

S1 Schematic illustration of the weight loss of lepidocrocite, as quantified by the stepwise method. S5

S2 Schematic illustration of the weight loss of lepidocrocite, as quantified by the tangential method. S5

S3 ICP-OES calibration curves for all measured Fe spectral lines employing a linear 8-point fit at 0.01, 0.10, 0.50, 1.00, 5.00, 10.00, 25.00 and 50.00 ppm. Fits of all spectral lines feature a coefficient of determination $R^2 \geq 0.999$. Figure S3a displays the full calibration range, whilst Figure S3b shows a zoomed-in section of the three lowest standards employed above the blank. Note that the highest estimated limit of quantitation (LOQ) of ~0.91 ppm at 259.940 nm as listed in Table S1 lies just below the lowest standard employed. S7

S4 Comparison of the total amount of dissolved iron over time at various pH, as determined via calibration at the emission lines of 235.350 and 259.940 nm. Note that the concentrations as predicted via interpolation at one spectral line are within the standard deviation of three independent measurements of the other one. S8

S5 X-ray diffractograms of pure iron (hydr)oxide reference standards 2 line ferrihydrite ($2l{-}Fe(OH)_3(s)$), lepidocrocite ($\gamma{-}FeOOH(s)$), goethite ($\alpha{-}FeOOH(s)$), hematite ($\alpha - Fe_2O_3(s)$) and magnetite ($\alpha - Fe_3O_4(s)$). S9

S6 Fe K-edge EXAFS $k^3\chi(\kappa)$ spectra of pure iron (hydr)oxide reference standards 2-line ferrihydrite ($2l{-}Fe(OH)_3(s)$), lepidocrocite ($\gamma{-}FeOOH(s)$), goethite ($\alpha{-}FeOOH(s)$), hematite ($\alpha{-}Fe_2O_3(s)$) and magnetite ($\alpha{-}Fe_3O_4(s)$). S10

S7 Time-dependent fractions of the respective reference solids obtained from LFC excluding lepidocrocite (Figure S7a) and including lepidocrocite (Figure S7b). Fits were achieved using the reference standards $2l{-}Fe(OH)_3(s)$, $\alpha{-}FeOOH(s)$ and $\gamma - FeOOH(s)$, and the fitting range was 2 to 9 Å^{-1}. S11

S8 Molar ratios of 2-line ferrihydrite Fe(t) relative to the initial iron concentration Fe_0 over time at various pH. S12
S9 Molar ratios of 2-line ferrihydrite Fe(t) relative to the initial iron concentration Fe_0 over time at various pH. S13

List of Tables

S1 LOD and LOQ in $\mu g\,L^{-1}$ and $\mu mol\,L^{-1}$ for the low-concentration element Fe, as determined by the recommendations of Caruso et al..[50] Concentrations are obtained by means of a linear 8-point interpolation containing 0.01, 0.10, 0.50, 1.00, 5.00, 10.00, 25.00 and 50.00 ppm of the measured elements. S6
S2 Elemental composition of the ICP calibration solutions used to determine the total amount dissolved aqueous iron. For all runs, the blank solution consisted of 2 wt.% HNO_3 in UPW. S8

Quantification of iron hydroxide phases by TGA

The thermal phase transformation of 2-line ferrihydrite to hematite involves the elimination of 3 equivalents of $H_2O(g)$ per equivalent of $Fe_2O_3(s)$ according to

$$2 \cdot 2l - Fe(OH)_3(s) \rightarrow \alpha - Fe_2O_3(s) + 3H_2O(g). \tag{1}$$

Similarly, the dehydration of goethite and lepidocrocite proceeds via the elimination of 1 equivalent of $H_2O(g)$

$$2 \cdot \alpha-,\ \gamma - FeOOH(s) \rightarrow \alpha - Fe_2O_3(s) + H_2O(g). \tag{2}$$

The stoichiometry of reactions 1 and 2 dictates that 3/2 and 1/2 equivalents of $H_2O(g)$ are lost per unit of $2l - Fe(OH)_3(s)$ and $\alpha-,\ \gamma - FeOOH(s)$, respectively. Taking WL to be the weight loss of water in g and MW_{H_2O} to be the molecular weight of water in $g\,mol^{-1}$, the number of moles of $2l - Fe(OH)_3(s)$ required to produce WL grams of water via Reaction 1 is

$$n_{2l-Fe(OH)_3(s)} = WL \times \frac{1}{3/2 \cdot MW_{H_2O}}. \tag{3}$$

By analogous reasoning, the number of moles of $\alpha-,\ \gamma - FeOOH(s)$ required to produce WL grams of water via Reaction 2 is

$$n_{\alpha-,\ \gamma-FeOOH(s)} = WL \times \frac{1}{1/2 \cdot MW_{H_2O}}. \tag{4}$$

The weight loss of water may be computed either by stepwise integration of the DTG curve, as illustrated in Figure S1, or alternatively by the tangential method displayed in Figure S2. Stepwise methods quantify the weight loss by uncorrected integration of the DTG. In contrast, the tangential method assumes that weight changes previous to and after the DTG peak of interest continue linearly.[43]

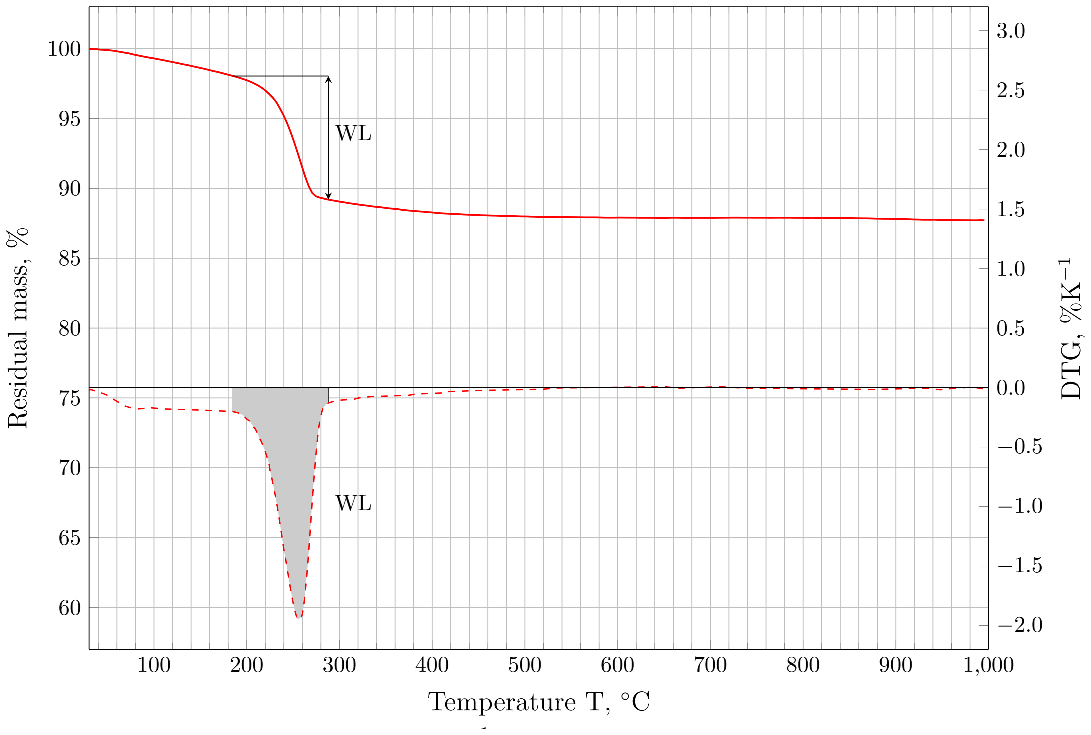

Figure S1: Schematic illustration of the weight loss of lepidocrocite, as quantified by the stepwise method.

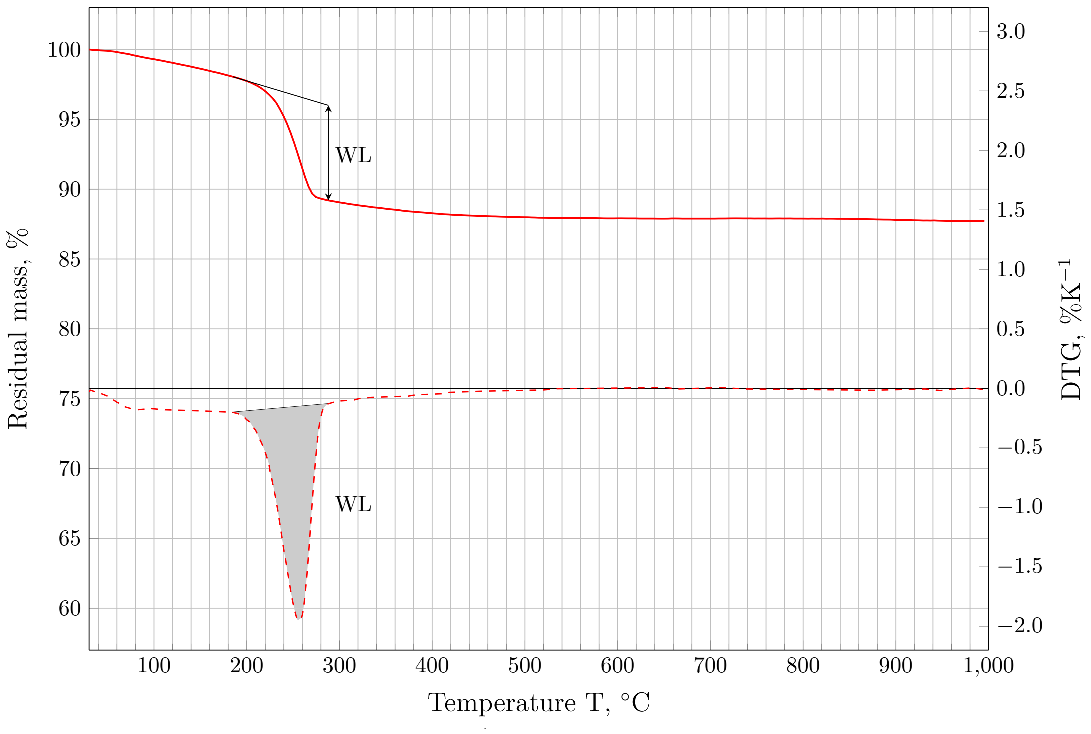

Figure S2: Schematic illustration of the weight loss of lepidocrocite, as quantified by the tangential method.

Calibration of ICP-OES

In line with the recommendations of of Caruso et al.,[50] the analyte's limit of detection (LOD) and quantitation (LOQ) were computed as three and ten times the standard deviation in the signal intensity of ten independently prepared blanks containing 280 $mg\,L^{-1}$ of Na, 200 $mg\,L^{-1}$ of K and S and 80 $mg\,L^{-1}$ of Ca. The resultant limits of detection (LOD) and quantitation (LOQ) are reported in Table S1. For the purpose of additional quality control, known concentrations of 0.1 mM and 0.3 mM of $FeCl_3 \cdot 6H_2O(cr)$ in 2 wt.% HNO_3 were analysed, and their concentration recalculated for all spectral lines considered. It was found that the measured concentrations differed from the expected ones by less than 1% at 234.350 and 259.940 nm, whilst the closeness of agreement was significantly lower for the remaining spectral lines, exceeding 1 and 4 % at 238.204 and 239.563 nm, respectively. The highest LOQ, i.e. 9.1 $\mu g\,L^{-1}$ at 259.940 nm was considered to be the global limit of quantitation. Correspondingly, the lowest concentration standard was chosen to be just above the global LOQ at 10 $\mu g\,L^{-1}$, as reported in Figure S3 and Table S2.

Table S1: LOD and LOQ in $\mu g\,L^{-1}$ and $\mu mol\,L^{-1}$ for the low-concentration element Fe, as determined by the recommendations of Caruso et al..[50] Concentrations are obtained by means of a linear 8-point interpolation containing 0.01, 0.10, 0.50, 1.00, 5.00, 10.00, 25.00 and 50.00 ppm of the measured elements.

Element	Spectral line, nm	SD	LOD		LOQ	
		(ppm)	($\mu g\,L^{-1}$)	($\mu mol\,L^{-1}$)	($\mu g\,L^{-1}$)	($\mu mol\,L^{-1}$)
Fe	234.350	$2.961 \cdot 10^{-4}$	0.888	0.016	2.961	0.053
	238.204	$4.726 \cdot 10^{-4}$	1.418	0.025	4.726	0.085
	239.563	$4.348 \cdot 10^{-4}$	1.304	0.023	4.348	0.078
	259.940	$9.081 \cdot 10^{-4}$	2.724	0.049	9.081	0.163

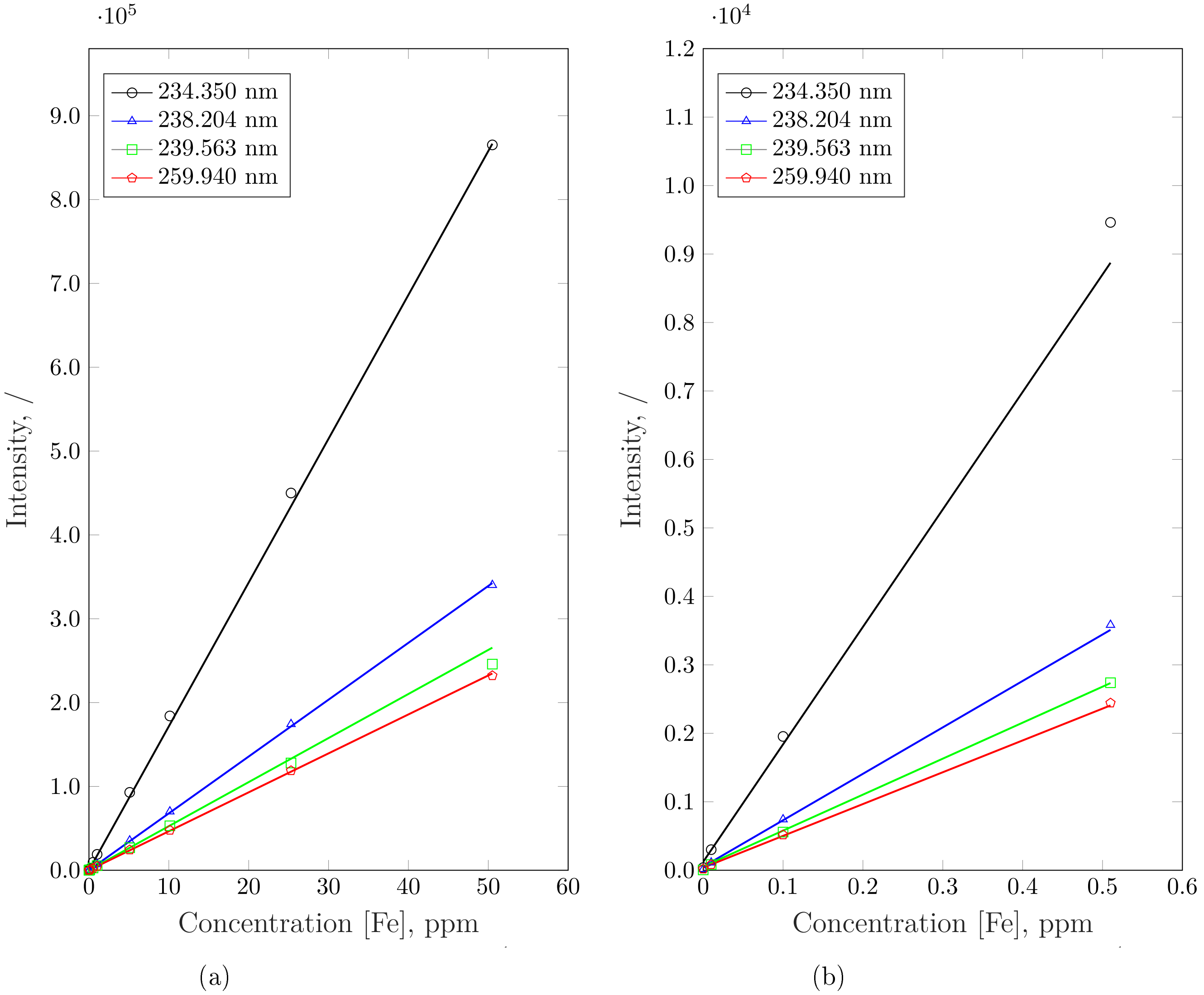

Figure S3: ICP-OES calibration curves for all measured Fe spectral lines employing a linear 8-point fit at 0.01, 0.10, 0.50, 1.00, 5.00, 10.00, 25.00 and 50.00 ppm. Fits of all spectral lines feature a coefficient of determination $R^2 \geq 0.999$. Figure S3a displays the full calibration range, whilst Figure S3b shows a zoomed-in section of the three lowest standards employed above the blank. Note that the highest estimated limit of quantitation (LOQ) of ~0.91 ppm at 259.940 nm as listed in Table S1 lies just below the lowest standard employed.

Table S2: Elemental composition of the ICP calibration solutions used to determine the total amount dissolved aqueous iron. For all runs, the blank solution consisted of 2 wt.% HNO_3 in UPW.

Standard	Element, ppm								
Number	Na	K	Ca	Mg	Al	Fe	S	Si	P
8	50.5506	50.2203	50.3531	50.4780	20.2052	50.3020	50.2344	50.3595	50.3269
7	25.3110	25.1456	25.2121	25.2747	10.1169	25.1865	25.1527	25.2153	25.1990
6	10.1346	10.0684	10.0950	10.1201	4.0508	10.0848	10.0712	10.0963	10.0898
5	5.0766	5.0435	5.0568	5.0693	2.0291	5.0517	5.0449	5.0574	5.0542
4	1.0239	1.0172	1.0199	1.0224	0.4093	1.0188	1.0175	1.0200	1.0194
3	0.5096	0.5063	0.5076	0.5089	0.2037	0.5071	0.5064	0.5077	0.5640
2	0.1017	0.1010	0.1013	0.1016	0.0407	0.1012	0.1011	0.1013	0.1013
1	0.0101	0.0101	0.0101	0.0102	0.0041	0.0101	0.0101	0.0101	0.0101

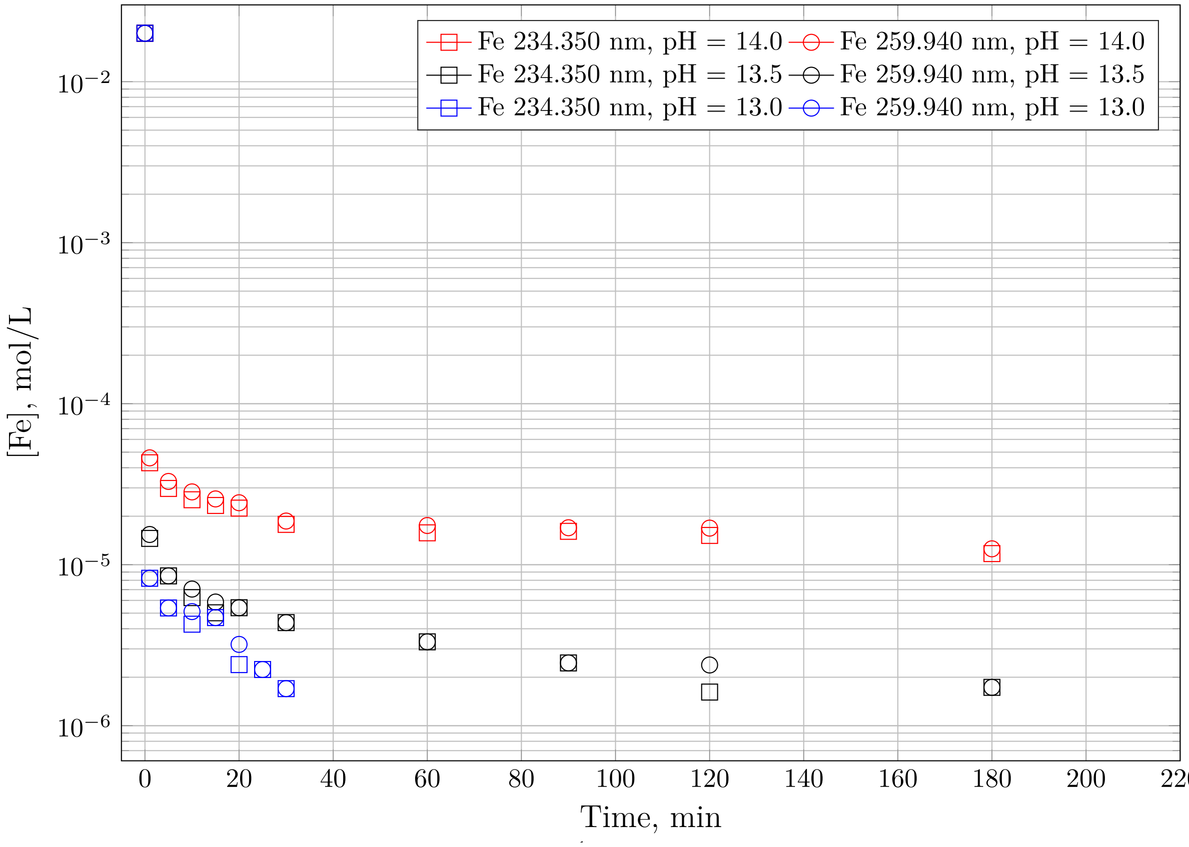

Figure S4: Comparison of the total amount of dissolved iron over time at various pH, as determined via calibration at the emission lines of 235.350 and 259.940 nm. Note that the concentrations as predicted via interpolation at one spectral line are within the standard deviation of three independent measurements of the other one.

XRD and XAS fitting and reference spectra

The XRD primary beam divergent slit distance from the sample, irradiated length, and secondary beam anti-scatter slit distance to the sample were equal to 180, 15, and 270 mm, respectively. Both primary and secondary beams feature a Soller slit opening angle of 2.3°. The primary beam axial mask used is 10 mm wide located at a distance of 110 mm. Sample holders measure 25 mm in diameter and have a linear absorption coefficient of 50 cm^{-1}.

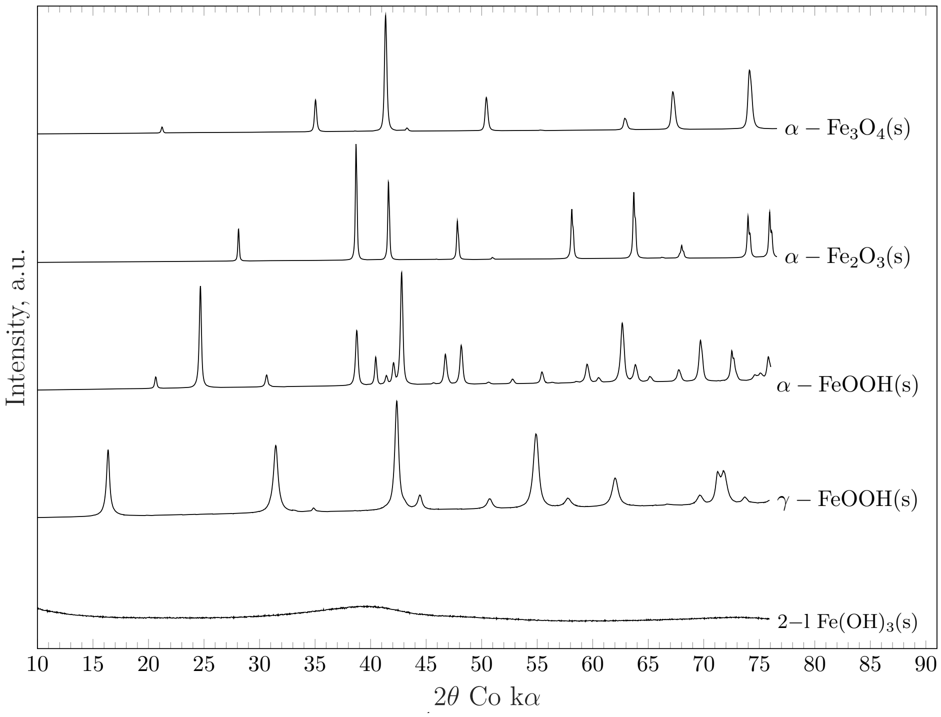

Figure S5: X-ray diffractograms of pure iron (hydr)oxide reference standards 2 line ferrihydrite (2l–$Fe(OH)_3(s)$), lepidocrocite ($\gamma - FeOOH(s)$), goethite ($\alpha - FeOOH(s)$), hematite ($\alpha - Fe_2O_3(s)$) and magnetite ($\alpha - Fe_3O_4(s)$).

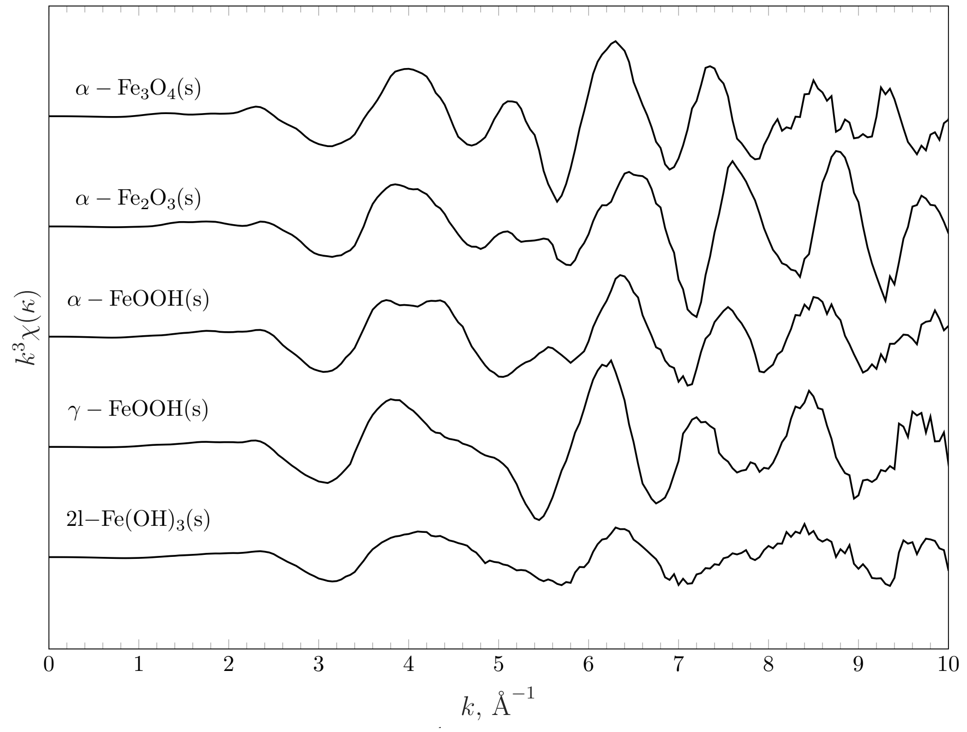

Figure S6: Fe K-edge EXAFS $k^3\chi(\kappa)$ spectra of pure iron (hydr)oxide reference standards 2-line ferrihydrite ($2l{-}Fe(OH)_3(s)$), lepidocrocite ($\gamma{-}FeOOH(s)$), goethite ($\alpha{-}FeOOH(s)$), hematite ($\alpha{-}Fe_2O_3(s)$) and magnetite ($\alpha{-}Fe_3O_4(s)$).

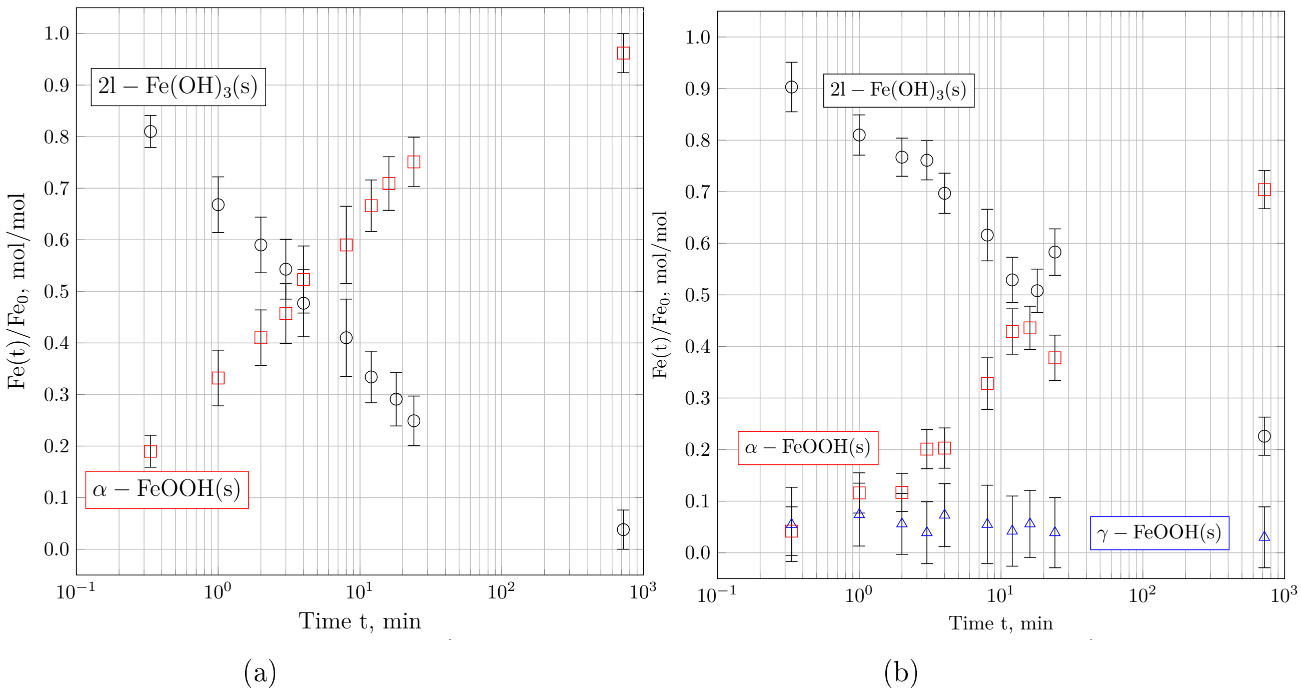

Figure S7: Time-dependent fractions of the respective reference solids obtained from LFC excluding lepidocrocite (Figure S7a) and including lepidocrocite (Figure S7b). Fits were achieved using the reference standards 2l$-Fe(OH)_3$(s), α − FeOOH(s) and γ − FeOOH(s), and the fitting range was 2 to 9 $Å^{-1}$.

First order fitting results

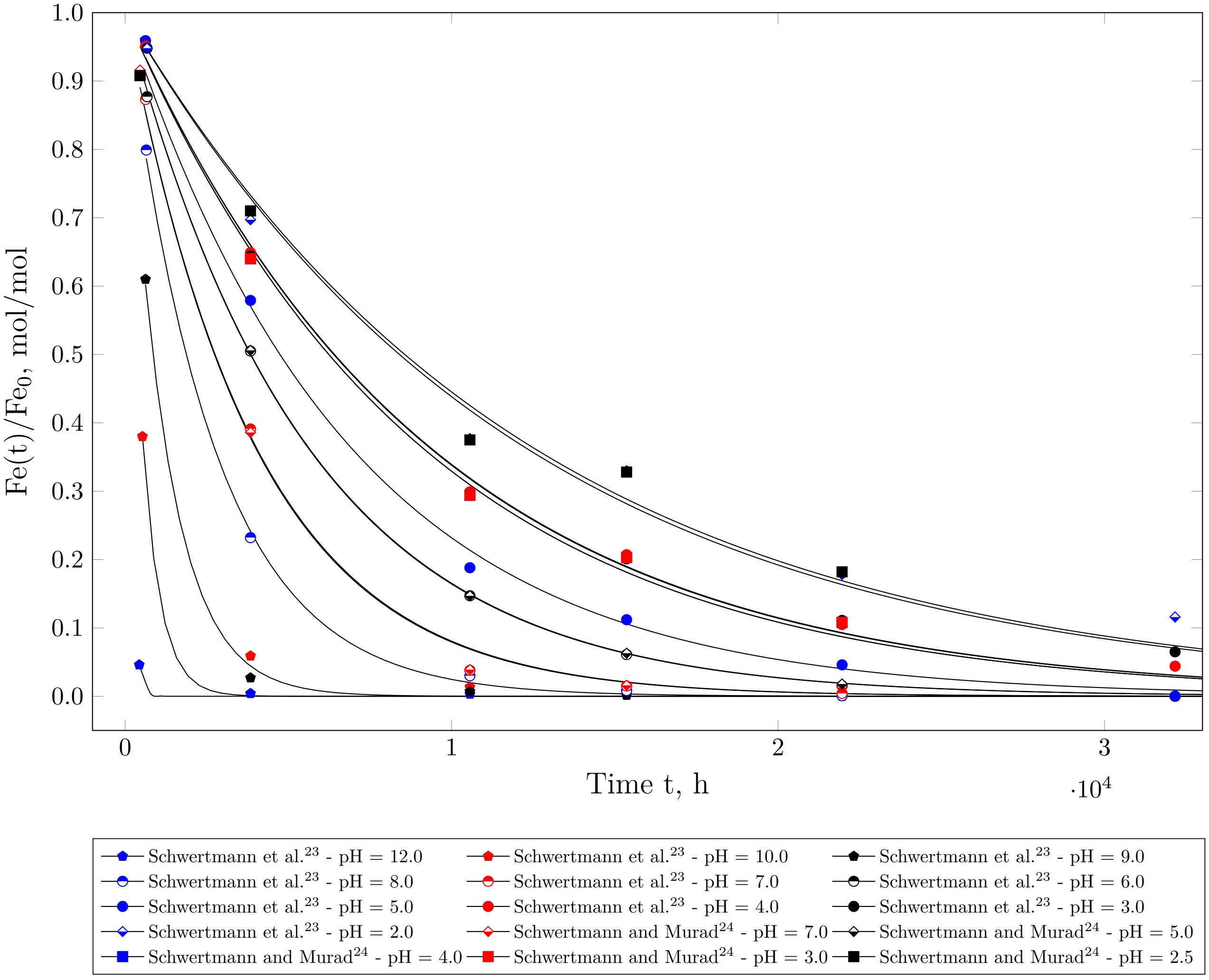

Figure S8: Molar ratios of 2-line ferrihydrite Fe(t) relative to the initial iron concentration Fe_0 over time at various pH.

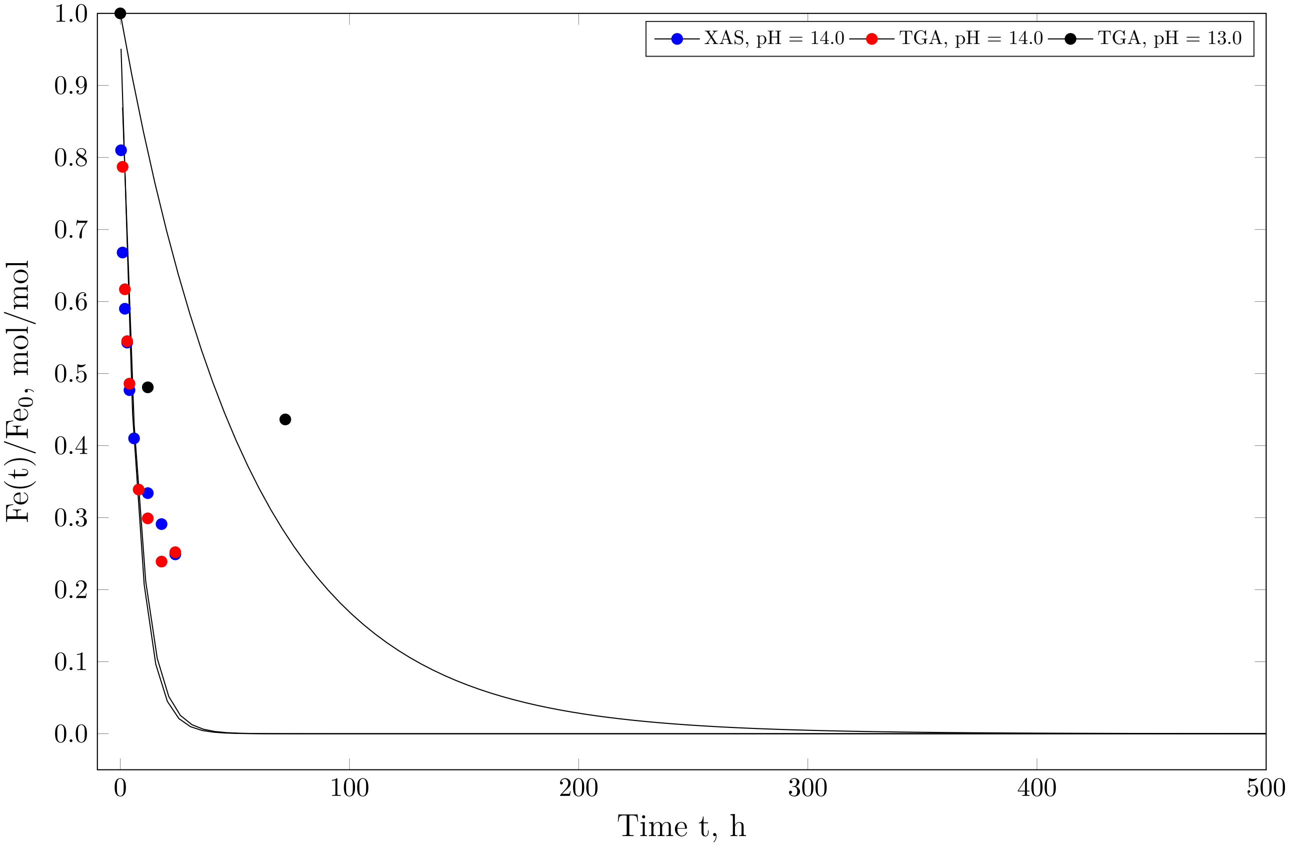

Figure S9: Molar ratios of 2-line ferrihydrite Fe(t) relative to the initial iron concentration Fe_0 over time at various pH.
